# Novel quorum sensing inhibitor Echinatin as an antibacterial synergist against *Escherichia coli*

**DOI:** 10.3389/fmicb.2022.1003692

**Published:** 2022-11-01

**Authors:** Yu-Bin Bai, Meng-Yan Shi, Wei-Wei Wang, Ling-Yu Wu, Yu-Ting Bai, Bing Li, Xu-Zheng Zhou, Ji-Yu Zhang

**Affiliations:** ^1^Key Laboratory of New Animal Drug Project of Gansu Province, Lanzhou, China; ^2^Key Laboratory of Veterinary Pharmaceutical Development, Ministry of Agriculture, Lanzhou, China; ^3^Lanzhou Institute of Husbandry and Pharmaceutical Sciences, Chinese Academy of Agricultural Sciences, Lanzhou, China

**Keywords:** *Escherichia coli*, quorum sensing, inhibitor, Echinatin, biofilm, *EPS*, motility, antibacterial synergist

## Abstract

A new antibacterial strategy based on inhibiting bacterial quorum sensing (QS) has emerged as a promising method of attenuating bacterial pathogenicity and preventing bacterial resistance to antibiotics. In this study, we screened Echinatin (Ech) with high-efficiency anti-QS from 13 flavonoids through the AI-2 bioluminescence assay. Additionally, crystal violet (CV) staining combined with confocal laser scanning microscopy (CLSM) was used to evaluate the effect of anti-biofilm against *Escherichia coli (E. coli)*. Further, the antibacterial synergistic effect of Ech and marketed antibiotics were measured by broth dilution and Alamar Blue Assay. It was found that Ech interfered with the phenotype of QS, including biofilm formation, exopolysaccharide (*EPS*) production, and motility, without affecting bacterial growth and metabolic activity. Moreover, qRT-PCR exhibited that Ech significantly reduced the expression of QS-regulated genes (*luxS*, *pfs*, *lsrB*, *lsrK*, *lsrR*, *flhC*, *flhD*, *fliC*, *csgD*, and *stx2*). More important, Ech with currently marketed colistin antibiotics (including colistin B and colistin E) showed significantly synergistically increased antibacterial activity in overcoming antibiotic resistance of *E. coli*. In summary, these results suggested the potent anti-QS and novel antibacterial synergist candidate of Ech for treating *E. coli* infections.

## Introduction

As one of the pathogenically versatile bacterial organisms, *E. coli* can cause various infections, including diarrhea, urinary tract infections, sepsis, and hemolytic-uremic syndrome (Riley, 2020). Pathogenic *E. coli* causes great economic losses to animal and poultry industries, as well as a serious threat to human health. For example, millions of dollars are lost each year due to Avian pathogenic *E. coli* (APEC) infections (Lutful Kabir, 2010). In the United States, *E. coli* O157:H7 is estimated to cause 95,000 illnesses a year (Scallan et al., 2011; Beshearse et al., 2021), and Shiga-producing *E. coli* (STEC) causes 5,960 infections annually (Hale et al., 2012). Antimicrobial resistance has become a worldwide concern and an increasing threat to human and animal health (Hawkey, 2008). Currently, most antibacterial compounds target the basic physiological processes of bacteria, which increases the likelihood of bacteria developing resistance to multiple drugs (Munguia and Nizet, 2017). Thus, new therapeutic strategies are urgently needed for treating multidrug-resistant pathogen infections.

At present, alternative approaches to antimicrobial therapy have focused on inhibiting the virulence factors of bacterial pathogens (Dickey et al., 2017; Buroni and Chiarelli, 2020; Loubet et al., 2020; Piewngam et al., 2020; Silva et al., 2020). QS is a cellular mechanism mediated by autoinducers, which allows bacteria to organize behavior depending on their density (Ng and Bassler, 2009). Interference with QS systems does not exert selection pressure on bacteria compared with antibiotics thus reducing the emergence and spread of resistant mutants (Sully et al., 2014; Quave et al., 2015; Ning et al., 2021). QS is a process that involves bacteria communicating with signaling molecules called autoinducers (AIs). There are several types of AI molecules, including diffusible signaling factors (DSFs), autoinducer-2 (AI-2), indole, and Acyl-homoserine lactones (AHLs, AI-1), etc. (Guo et al., 2013; Whiteley et al., 2017, 2018). QS systems function based on cell density, which increases the concentration of AI as cell density increases. Upon reaching a certain level of concentration of AI, signaling is activated that modulates the expression of genes related to bacterial physiology, biofilm formation, motility, and virulence (Papenfort and Bassler, 2016). Antibacterial strategies based on inhibiting bacterial QS have emerged as a new promising method of preventing bacterial resistance to antibiotics, as well as inhibiting the expression of virulence factors (Manner and Fallarero, 2018; Wang et al., 2019; Minich et al., 2022).

AI-2 has been considered to be a “universal” signaling molecule involved in bacterial communication at the inter-and intra-species level, which is widely found in Gram-negative and Gram-positive bacteria. For most bacteria, the regulatory function of the AI-2 QS system is mainly reflected in four aspects, including bacterial virulence, biofilm, motility, and other functions (Bassler, 2002; Sturme et al., 2002; Reading and Sperandio, 2006; Choudhary and Schmidt-Dannert, 2010). For example, AI-2 could influence the production of virulence factors and the formation of biofilm in *E. coli*, *Salmonella*, and *S. suis* (Kendall et al., 2007; Ju et al., 2018; Sun et al., 2020; Li et al., 2021). The inhibition of AI-2 can effectively reduce bacterial virulence, biofilm formation, and bacterial resistance, which can be used to replace antibiotics. In this context, it’s reported that plant-derived compounds with diverse structures have been widely investigated as AI-2 QS inhibitors (Karnjana et al., 2020; Li et al., 2021; Meng et al., 2022). Ech, a flavonoid isolated from glycyrrhiza, had antioxidant, antitumor, anti-virus, and other biological activities (Ji et al., 2016; Zhu et al., 2018; Kwak et al., 2019; Hu et al., 2021; Ran et al., 2021). Despite many pharmacologic investigations, there have been no reports on the anti-QS activity of Ech.

AI-2 bioluminescence assay (*Vibrio harveyi* (*V. harveyi*) BB170 bioluminescence assay) is the most commonly used method to detect AI-2 signal molecules. The bioluminescence reporter strain *V. harveyi* BB170 (luxN:: Tn5) is a mutant strain, whose fluorescence is only regulated by AI-2 signal molecule (Bassler et al., 1994, 1997; Surette et al., 1999). Therefore, the inhibitory effect of the inhibitors on *E. coli* AI-2 production can be judged by the intensity of *V. harveyi* bioluminescence. Here, based on *V. harveyi* BB170 bioluminescence assay, 13 natural product compounds that inhibit AI-2 production were screened and evaluated. Notably, we found that one of the compounds, Ech, effectively blocks *E. coli* QS. Furthermore, the study examined the antibiofilm and antivirulence properties of Ech against *E. coli* and evaluated the synergistic effects of combining Ech with antibiotics.

## Materials and methods

### Natural product compounds

Echinatin, Aloeemodin, Loureirin B, Cardamonin, Cynaroside, Artemetin, Neosperidin dihydrochalcone, Hesperetin, and Vitexin used in the study were obtained from Shanghai Yuanye Bio-Technology Co., Ltd. (Shanghai, China). Phloretin was purchased from Macklin Inc. (Shanghai, China). Scutellarin and Acacetin were obtained from MCE (Shanghai, China). Gentisin was obtained from ChemFaces (Wuhan, China). Dimethylsulfoxide (DMSO, Sigma) was used to dissolve all compounds to the concentration of 40 mM.

### Bacterial strains and cells

*Escherichia coli* O157:H7 (ATCC 43895) was purchased from Beina Chuanglian Biotechnology Research Institute (Beijing, China). Clinical strains *E. coli* O101、*E. coli* C83654、*E. coli* O149、*E. coli* XJ24、*E. coli* KD-13-1 were isolated and maintained in our laboratory. Luria-Bertani (LB, HuanKai Microbial, Guangdong, China) and Luria-Bertani agar (LA, HuanKai Microbial, Guangdong, China) medium were used to cultivate all *E. coli* strains. *V. harveyi* BB170 and *V. harveyi* BB152 were kindly provided by Researcher Han Xiangan (Shanghai Veterinary Research Institute, Chinese Academy of Agricultural Sciences). AB medium supplemented with 1 mM L-arginine, 1% glycerol, and 10 mM Phosphate buffer (pH = 7.2) for culturing *V. harveyi*.

Caco-2 cell lines were obtained from ATCC and cultured under standard conditions containing MEM medium (Gibco, Grand Island, NY, United States) supplemented with 20% FBS (Gibco, Grand Island, NY, USA), 1% non-essential amino acids (Gibco, Grand Island, NY, USA), 10 mM HEPES (Solarbio, Beijing, China), 1 mM L-glutamine (Gibco, Grand Island, NY, USA), and 1 mM sodium pyruvate (Gibco, Grand Island, NY, United States).

### Anti-QS inhibitor screening

AI-2 bioluminescence assay was used to screen QS inhibitors as described previously (Bassler et al., 1994; Taga and Xavier, 2011) with minor modifications. Briefly, *E. coli* O157:H7 was cultured for 16 h with 50 μM of natural product compounds and centrifuged at 12000 × g for 5 min. Cell-free supernatant was collected in a 0.22 μm filter. The bioluminescence reporter strain *V. harveyi* BB170 grew in AB medium to 1.0 ~ 1.1 of OD_600nm_ at 30°C under shaking and then diluted at 1:2500 with fresh AB medium. Twenty micro liter of cell-free supernatant mixed with 180 μl of *V. harveyi* BB170 culture in black 96-well plates (Jingan, Shanghai, China) and incubated for 3.5 h at 30°C in the dark. A multipurpose microplate reader (Enspire; PerkinElmer, USA) was used to measure bioluminescence. Cell-free supernatants of *V. harveyi* BB152 overnight cultures were used as control. Further study was conducted on the compound with the highest AI-2 inhibition. We also determined the IC50 for AI-2 inhibition with the selected compound with the same procedure described above.

### Cytotoxicity

Ech’s toxicity was evaluated in Caco-2 cells using the CCK-8 assay. Caco-2 cells (10^5^ cells/mL) were plated in a 96-well plate with 5% CO_2_ for 24 h at 37°C. The culture medium was then replaced by different concentrations of Ech (6.25, 12.5, 25, 50, 100, 200, 400 μM) for 24 h. After incubation, the plate was incubated at 37°C for an additional hour with 10 μl of CCK-8 (MCE, China). The absorbance was measured at 450 nm using Multiskan Go Reader (Thermo Fisher Scientific, United States).

### Growth and metabolic activity

As described previously, the growth ability of *E. coli* was studied by the broth dilution method (CLSI, 2018; Swetha et al., 2019). In brief, *E. coli* O157:H7 bacterial suspension (OD_600_ = 0.01) with various concentration of Ech (6.25, 12.5, 25, 50, 100, 200 μM) was seeded into 96-well plate and incubated for 24 h at 37°C. The Multiskan Go Reader (Thermo Fisher Scientific, United States) was used to measure the absorbance at 600 nm.

Metabolic activity was measured with an Alamar Blue assay (Swetha et al., 2019). *E. coli* O157:H7 bacterial suspension (OD_600_ = 0.01) with various concentrations of Ech (6.25, 12.5, 25, 50, 100, 200 μM) was seeded into 12-well plate and incubated for 24 h at 37°C. Cells from each well were harvested at 10000 × g for 5 min and then washed twice using PBS (pH = 7.2). Metabolic activity was measured using Alamar Blue assay according to the manufacturer’s prescribed protocol (Invitrogen™, Thermo Fisher Scientific, USA). PBS containing only AB dye was considered a blank. The metabolic activity was calculated based on the absorbance at 570 nm and 600 nm using the following formula (Swetha et al., 2019):


$$\mathrm{Metabolic}\;\mathrm{activity}\left(\%\right)=\frac{\left(\begin{array}{l} \left(E_{oxi\left(OD570\right)}\times T_{OD570}\right)\\ -\left(E_{oxi\left(OD600\right)}\times T_{OD600}\right) \end{array}\right)}{\left(\begin{array}{l} \left(E_{\mathrm{red}\left(OD570\right)}\times B_{OD570}\right)\\ -\left(E_{\mathrm{red}\left(OD600\right)}\times B_{OD600}\right) \end{array}\right)}\times 100\%$$

Eoxi (OD570) – extinction coefficient in oxidized form of AB at 570 nm = 80,586;

Ered (OD570) – extinction coefficient in reduced form of AB at 570 nm = 155,677;

Eoxi (OD600) – extinction coefficient in oxidized form of AB at 600 nm = 117,216;

Ered (OD600) – extinction coefficient in reduced form of AB at 570 nm = 14,652;

B-blank; T-samples.

### Biofilm assay

#### CV staining

The formation of biofilms was assessed using CV staining based on previous study (O’Toole, 2011) with slight modification. In brief, *E. coli* O157:H7 bacterial suspension (OD_600_ = 0.01) was cultured in LB medium at the various concentrations of Ech (6.25, 12.5, 25, 50, 100, 200 μM) in a 96-well plate (Corning Costar® 3,599, Corning, NY, United States) for 24 h at 37°C. The plate was washed three times with PBS (pH = 7.2) and then fixed for 1 h at 60°C. Methanol was used to fix the cells and 0.1% CV was used to stain them for 30 min. Next, the CV was rinsed with distilled water and dried under heat. Finally, the CV attached to wells was dissolved in 95% ethanol and then measured the absorbance at 570 nm using Multiskan Go Reader (Thermo Fisher Scientific, United States).

#### Confocal laser scanning microscopy

Biofilm formation of *E. coli* was determined using CLSM according to previous study (Zhang et al., 2020) with slight modification. In brief, *E. coli* O157:H7 bacterial suspension (OD_600_ = 0.01) supplemented with different concentrations of Ech (12.5, 25, 50 μM) was seeded into a 6-well plate with coverslips and incubated for 24 h at 37°C. The suspensions were removed, and the wells were washed with PBS (pH = 7.2). The biofilm was stained using BacLight Live/Dead viability kit (L7012, Invitrogen™, Thermo Fisher Scientific, United States) according to the procedure and observed by CLSM (Zeiss LSM800, Zeiss, Tokyo, Japan).

### *EPS* production

According to the previous method, Ruthenium Red staining assessed *EPS* production (Adnan et al., 2020). Cell suspensions (10^6^ CFU/ml) of *E. coli* O157:H7 and different concentrations of Ech were cultured in a 96-well plate for 24 h at 37°C. The plate was washed with PBS (pH = 7.2), stained with 0.01% ruthenium red (Yuanye, Shanghai, China), and then incubated at 37°C for 1 h. 0.01% ruthenium red was used to fill the wells without biofilm was used as blank. 0.01% ruthenium red was used to fill the wells with biofilm and without Ech was used as a positive control. The absorbance was performed at 450 nm using Multiskan Go Reader after the liquid carrying the residual stain was transferred to new 96-well plates (Thermo Fisher Scientific, USA). *EPS* inhibition was calculated as follows formula (Adnan et al., 2020):


$$EPS\;\mathrm{inhibition}\left(\%\right)=\frac{AS-AP}{\mathrm{AB}-AP}\times 100$$

Whereas:

AB = absorbance of the blank.

AS = absorbance of the sample.

AP = absorbance of the positive control.

### Motility assay

According to the previous description, the motility of *E.coli* was performed (Vikram et al., 2013). Briefly, overnight *E. coli* O157:H7 was diluted to OD_600_ = 0.01, and then a semisolid agar media (0.3% LB agar) containing 12.5, 25, and 50 μM of Gin was used for the motility assay. One micro liter of the diluted bacterial solution was inserted into the middle of the plate. DMSO alone was used as the control. Halo zone diameters were measured after incubation for 16–18 h at 37°C to assess motility.

### qRT-PCR

qRT-PCR was used to measure Ech’s effect on QS-regulated, biofilm formation, motility, and virulence factor-related genes of *E. coli*. *Escherichia coli* O157:H7 was incubated in the 12-wells plate with and without Ech at 37°C for 24 h. Bacterial RNA Kit(Omega, USA) was used to extract total RNA. RNA concentration was determined by NanoDrop OneC spectrophotometer (Thermo Scientific, USA). PrimeScript™ RT reagent Kit with gDNA Eraser (TAKARA Corporation, Japan) was used to reverse transcribe RNA into cDNA. qRT -PCR was analyzed using TB Green® Premix Ex TaqTM II (Tli RNaseH Plus) (TAKARA Corporation, Japan). Based on the 2^−∆∆Ct^ method, the relative changes in gene expression levels were analyzed. The *gapA* gene was used as an internal control (Hu et al., 2013). This study used the primers listed in Supplementary Table 1.

### Antibacterial activity

Antibacterial activity was evaluated based on the previous method (Swetha et al., 2019; Liu et al., 2021) with some modifications. Briefly, *E. coli* O157:H7 and five clinical strains (*E. coli* O101, *E. coli* C83654, *E. coli* O149, *E. coli* XJ24, *E. coli* KD-13-1) bacterial suspensions (OD_600_ = 0.01) were mixed with antibiotics (1/2 MIC, 1/4 MIC, 1/8 MIC) with or without Ech (50 μM) at 37°C for 16–18 h. Antibacterial effects were assessed by metabolic activity with Alamar Blue assay. Tests were conducted in triplicate.

### Statistical analysis

The experiment was repeated three times with three replicates for each treatment, and data represent the mean ± SD. The significance of differences was evaluated with multiple t-tests for two groups or non-parametric one-way ANOVA for multiple groups using GraphPad Prism (GraphPad Prism 8; GraphPad). Where *p < 0.05; **p < 0.01; ***p < 0.001; ****p < 0.0001.

## Results

### Screening of QS inhibitors against *Escherichia coli*

**Figure 1:**
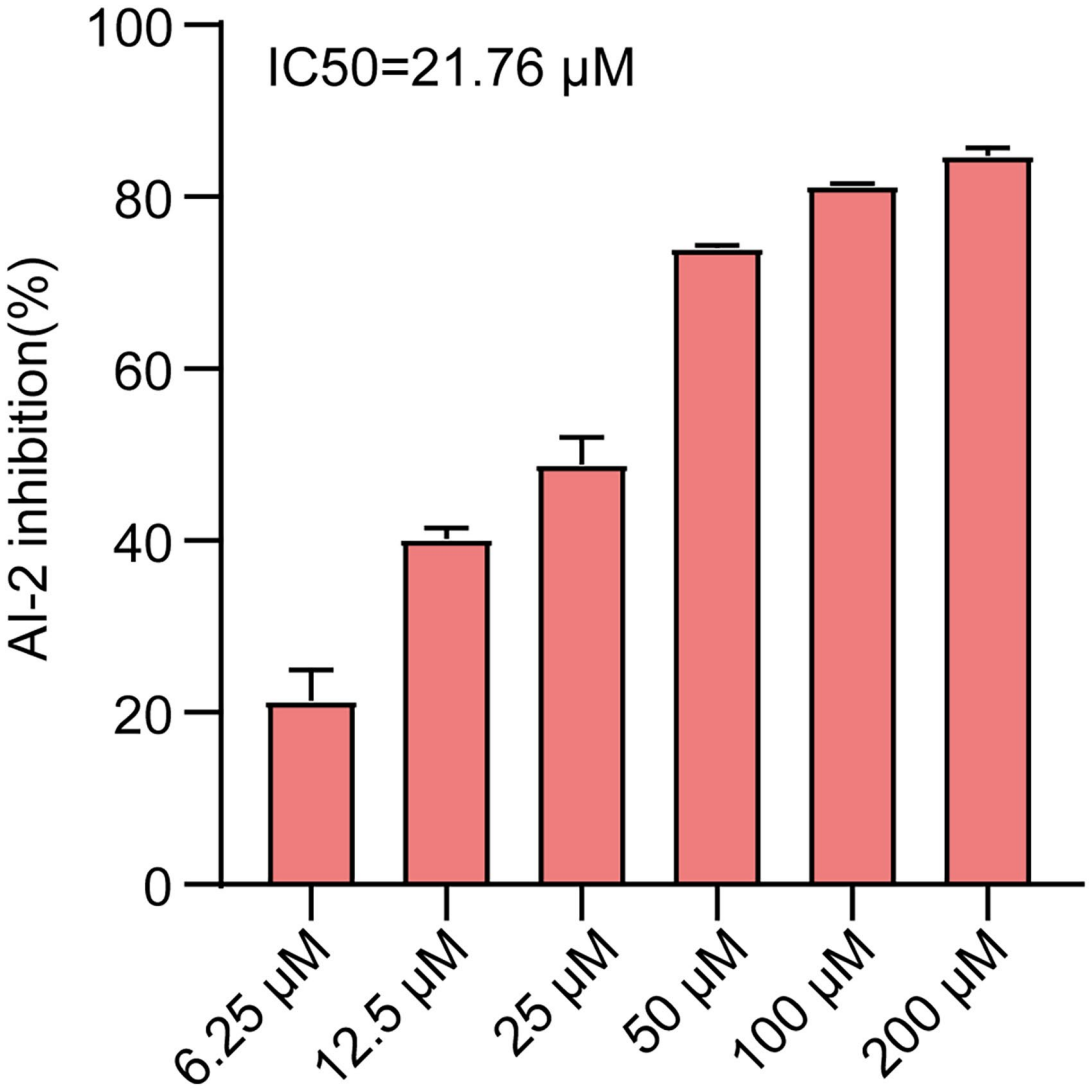
AI-2 inhibition of *E. coli* was treated with various concentrations of Ech (6.25, 12.5, 25, 50, 100, 200 μM) by AI-2 Bioluminescence assay and IC50 = 21.76 μM. All experiments were carried out in triplicate, and data represent the mean ± SD.

To identify new QS inhibitors, 13 flavonoid compounds from nature compounds were screened by the *V. harveyi* BB170 bioluminescence assay (Supplementary Table 2). The results showed that the QS inhibition rates of two compounds Ech and aloeemodin were greater than 70% at 50 μM. The QS inhibition rates of six compounds loureirin B, phloretin, cardamonin, neosperidin dihydrochalcone, cynaroside, and acacetin were between 60 and 70%, and five compounds scutellarin, artemetin, hesperetin, vitexin, and gentiin were less than 50%. In particular, Ech was the better anti-QS activity with an IC50 of 21.67 μM (Figure 1). Therefore, it was focused on during our study.

### Cytotoxicity of Ech On Caco-2

**Figure 2:**
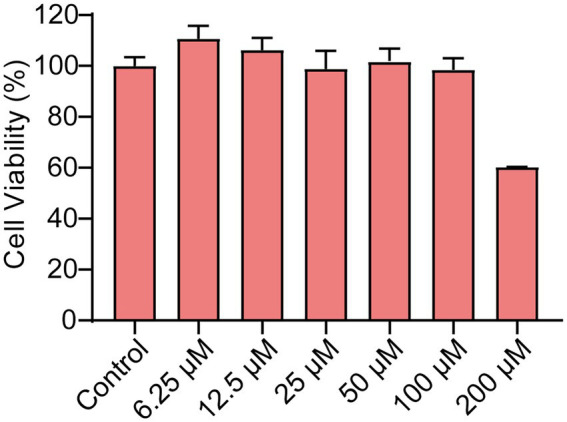
The Cytotoxicity of Ech in Caco-2 cells. The cells were treated with Ech at different concentrations (0, 6.25, 12.5, 25, 50, 100, and 200 μM) for 24 h. Data represent means ± SD of three experiments conducted in triplicate.

Ech’s cytotoxicity was assessed in the study to develop it as a safer alternative to antibiotics. The viability of Caco-2 cells was evaluated using CCK-8 assay after treatment with Ech at various concentrations (6.25, 12.5, 25, 50, 100, 200 μM). Compared with the control, Ech is non-toxic to Caco-2 cells at concentrations below 100 μM (Figure 2).

### Effects of Ech on growth and metabolic activity of *Escherichia coli*

**Figure 3:**
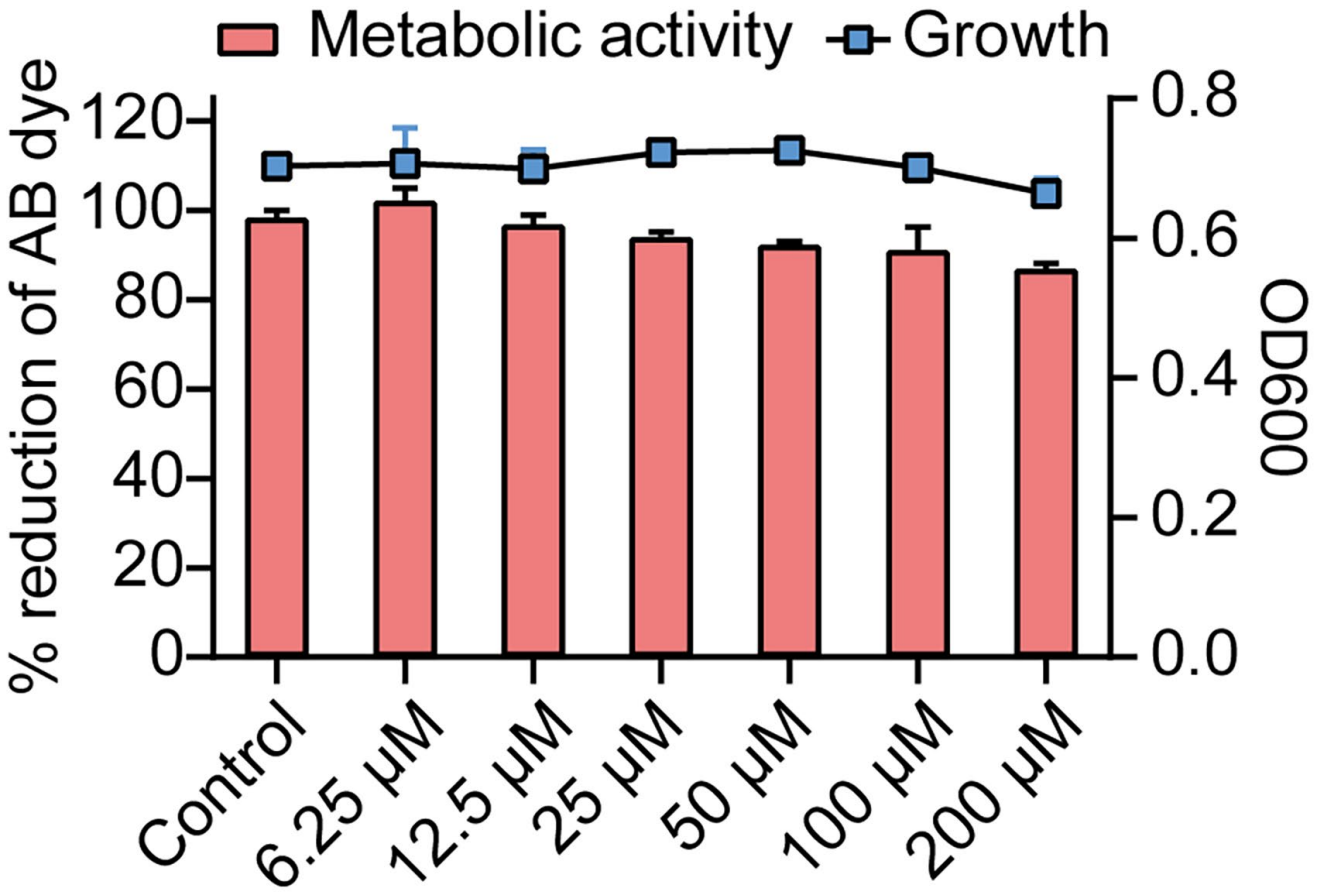
Growth and metabolic activity of *E. coli* in the presence of Ech. The line graph shows the growth of *E. coli* with Ech measured using a microbroth dilution assay. The bar graph shows the metabolic activity of *E. coli* based on the AB assay. Data represent means± SD of three experiments conducted in triplicate.

The effects of Ech on growth and metabolic activity were revealed by the microbroth dilution and Alamar Blue (AB) assay (Figure 3). Results showed that control and Ech treated cells showed no significant differences in the fluorescent intensity of AB dye. In addition, *E. coli* growth was not significantly different between the Ech treated sample and the control culture. These results showed the non-antibacterial effect of Ech.

### Effects of Ech on biofilm formation of *Escherichia coli*

**Figure 4:**
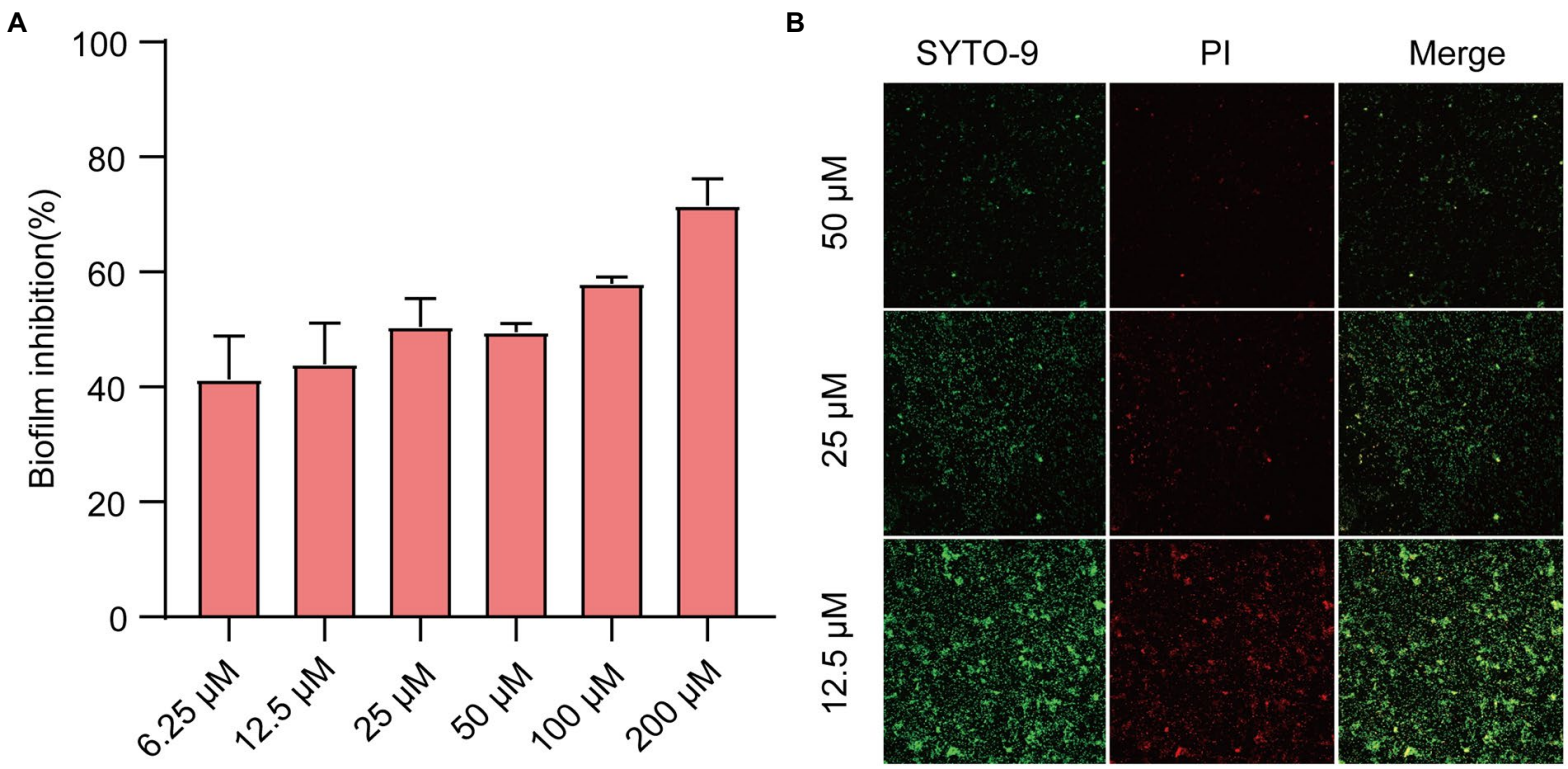
Effects of Ech on *E. coli* biofilm formation. **(A)** Biofilm formation inhibition at various concentrations of Ech (6.25, 12.5, 25, 50, 100, and 200 μM) for 24 h by CV staining. **(B)** Three-dimensional (3D) image of *E. coli* biofilm with Ech (12.5, 25, and 50 μM) for 24 h by CLSM. Error bars are mean ± SD.

Next, we assessed the effects of QS inhibitor on the formation of biofilm against *E. coli* by CV staining and CLSM. CV staining showed that Ech significantly inhibited the biofilm formation of *E. coli* in a dose-dependent manner (Figure 4A). More specifically, inhibition reached 40% at 6.25 μM of inhibitor concentration, while 71.54% inhibition was achieved at 200 μM. In addition, the antibiofilm of Ech was further verified by CLSM. As shown in Figure 4B, green fluorescence (living cells) and red fluorescence (death cells) showed significant decreases with increasing concentration, which proved that Ech could effectively inhibit the adhesion of *E. coli* and reduce biofilm formation.

### Effects of Ech on EPS production of *Escherichia coli*

**Figure 5:**
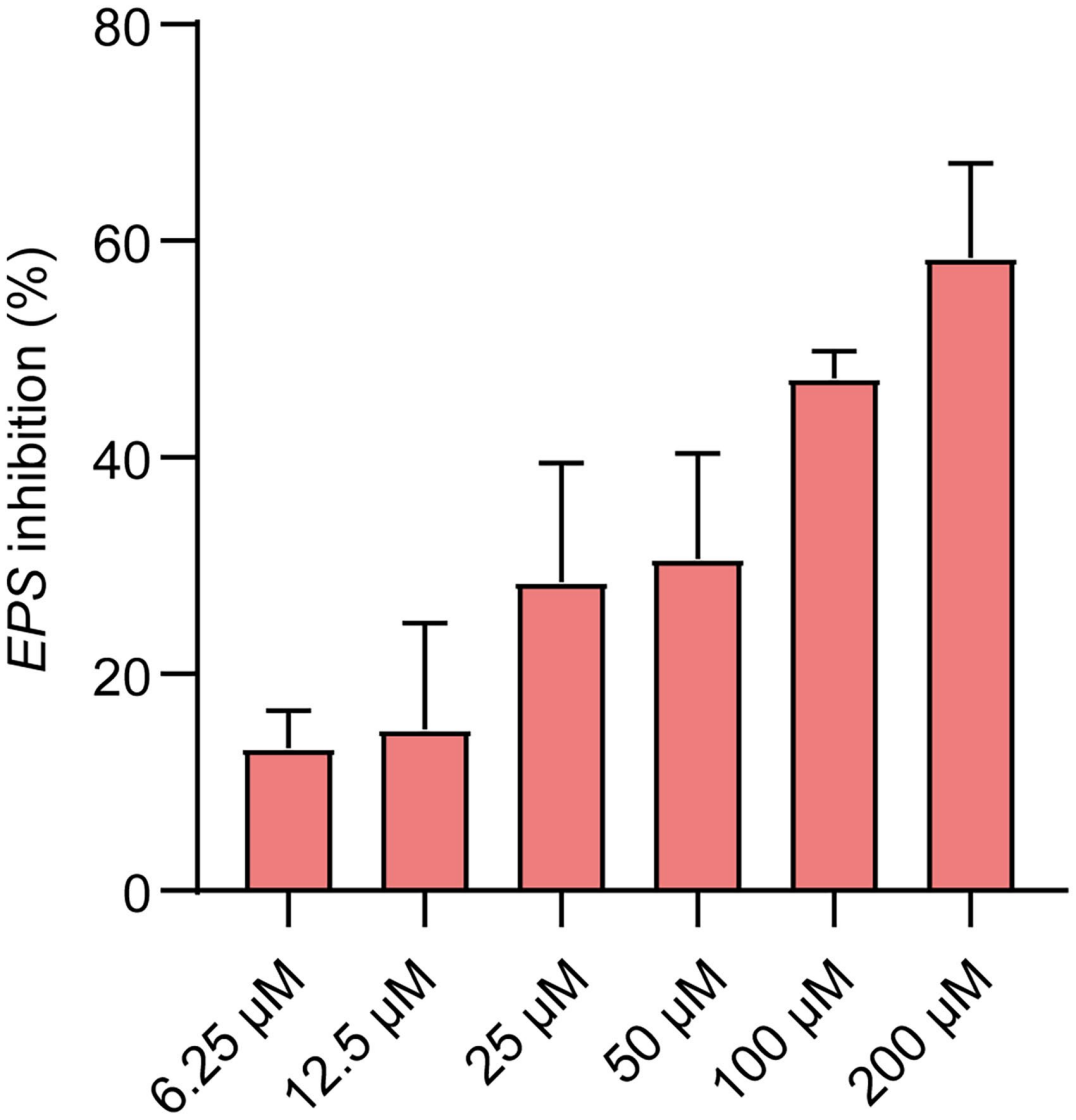
*EPS* inhibition (%) showed at the various concentrations of Ech (6.25, 12.5, 25, 50, 100, and 200 μM) for 24 h. Data represent means ± SD of three experiments conducted in triplicate.

In a biofilm matrix, *EPS* is among the most critical components (Branda et al., 2005). As was shown in Figure 5, Ech inhibited *EPS* production dose-dependently. The *EPS* inhibition reached 50% at the concentration of 100 μM. It was consistent with the results of CV staining. This result further confirms the inhibition of Ech on the formation of biofilm.

### Effects of Ech on the motility of *Escherichia coli*

**Figure 6:**
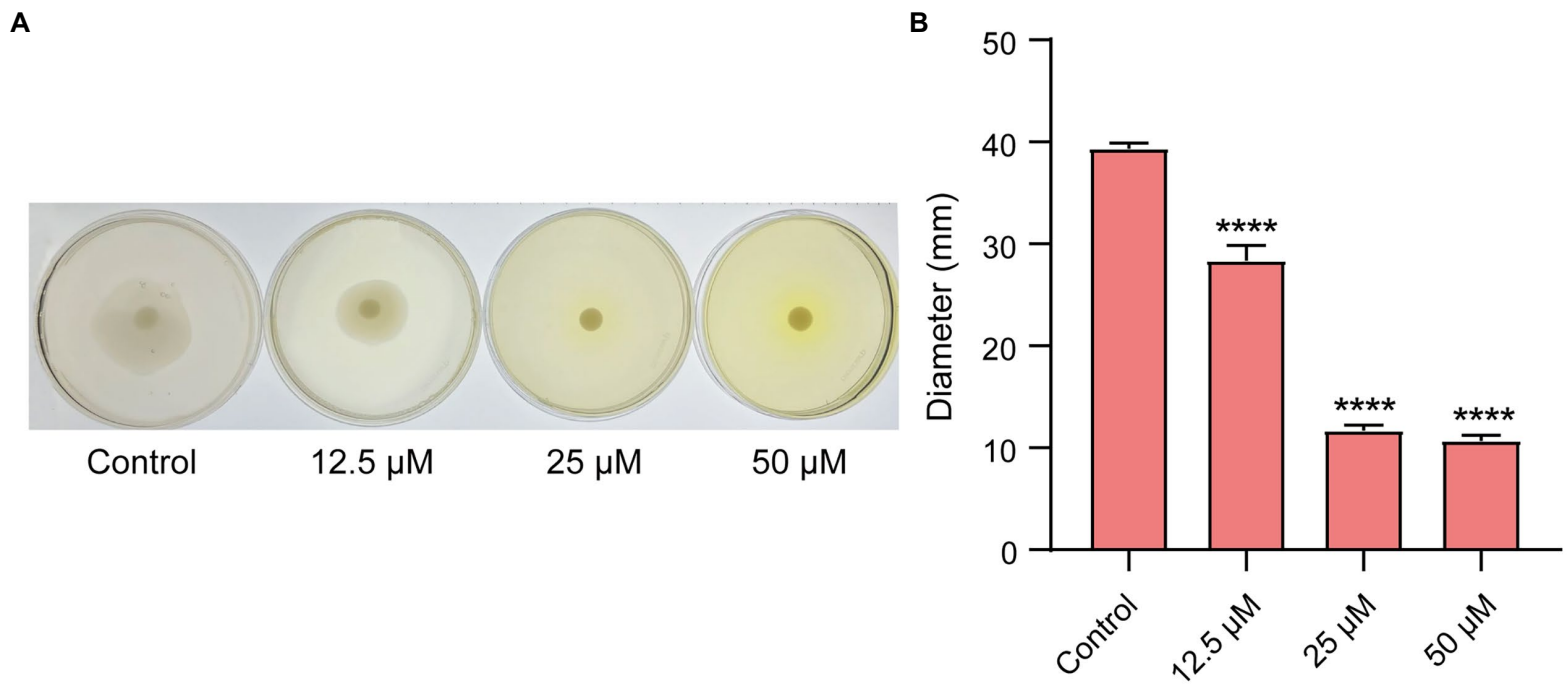
Motility inhibition of *E. coli* with Ech. **(A)** Images of motility following incubation with *E. coli* at the various concentration of Ech (0, 12.5, 25, and 50 μM). **(B)** Quantitative estimation of motility based on the halo zone’s diameter. **** = *p* < 0.0001.

Quorum-sensing controls the motility of *E.coli*. Figure 6A showed that QS inhibitor Ech reduced the motility in a dose-dependent. Further, we assessed the halo zone quantitatively (Figure 6B). Results showed that Ech significantly inhibited the motility of *E. coli* in comparison with the control group (*p* < 0.0001).

### Effect of Ech on the expression of QS-regulated genes of *Escherichia coli*

**Figure 7:**
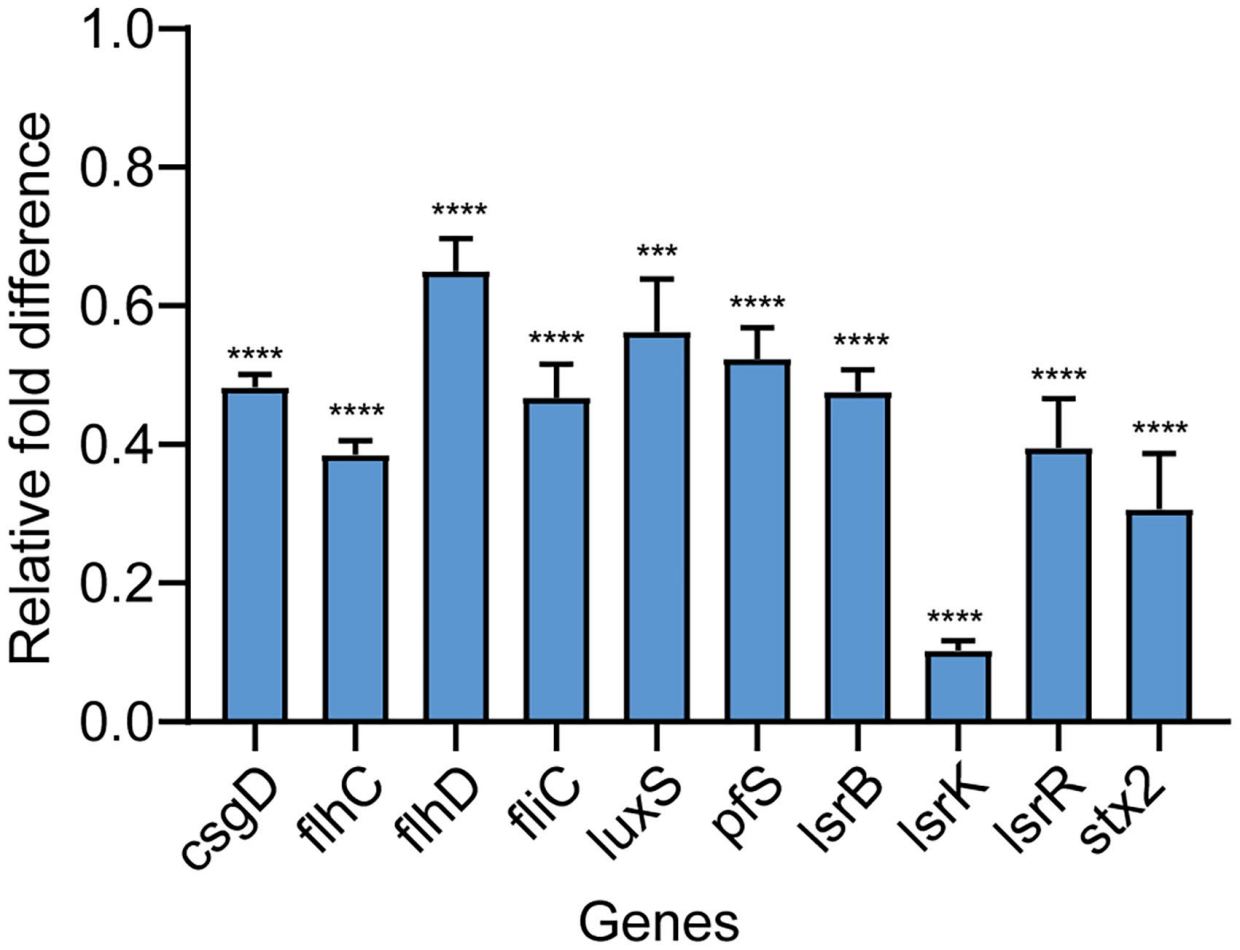
Effects of Ech on expression QS-regulated genes of *E. coli*. The qRT-PCR results showed significantly different nine genes (*csgD*, *flhC*, *flhD*, *fliC*, *luxS*, *pfs*, *lsrB*, *lsrK*, *lsrR*, and *stx2*) compared with the control. Data represent means ± SD of three experiments conducted in triplicate.

To further evaluate the potential molecular basis responsible for QS inhibition by Ech, we measured the expression of QS, motility, and biofilm genes by qRT - PCR. Ech reduced the expression of QS-regulated genes like *luxS*, *pfs*, *lsrB*, *lsrK*, and *lsrR* (43, 48, 52, 90, and 61% respectively), motility-regulated genes *flhC*, *flhD*, and *fliC* (61, 35, and 53%, respectively), biofilm-regulated genes *csgD* (52%), and virulence factor-regulated gene *stx2* (69%; Figure 7). The result demonstrated that Ech inhibited QS, biofilm formation, motility, and virulence factor production.

### Synergistic effects of Ech with antibiotics against *Escherichia coli*

**Figure 8:**
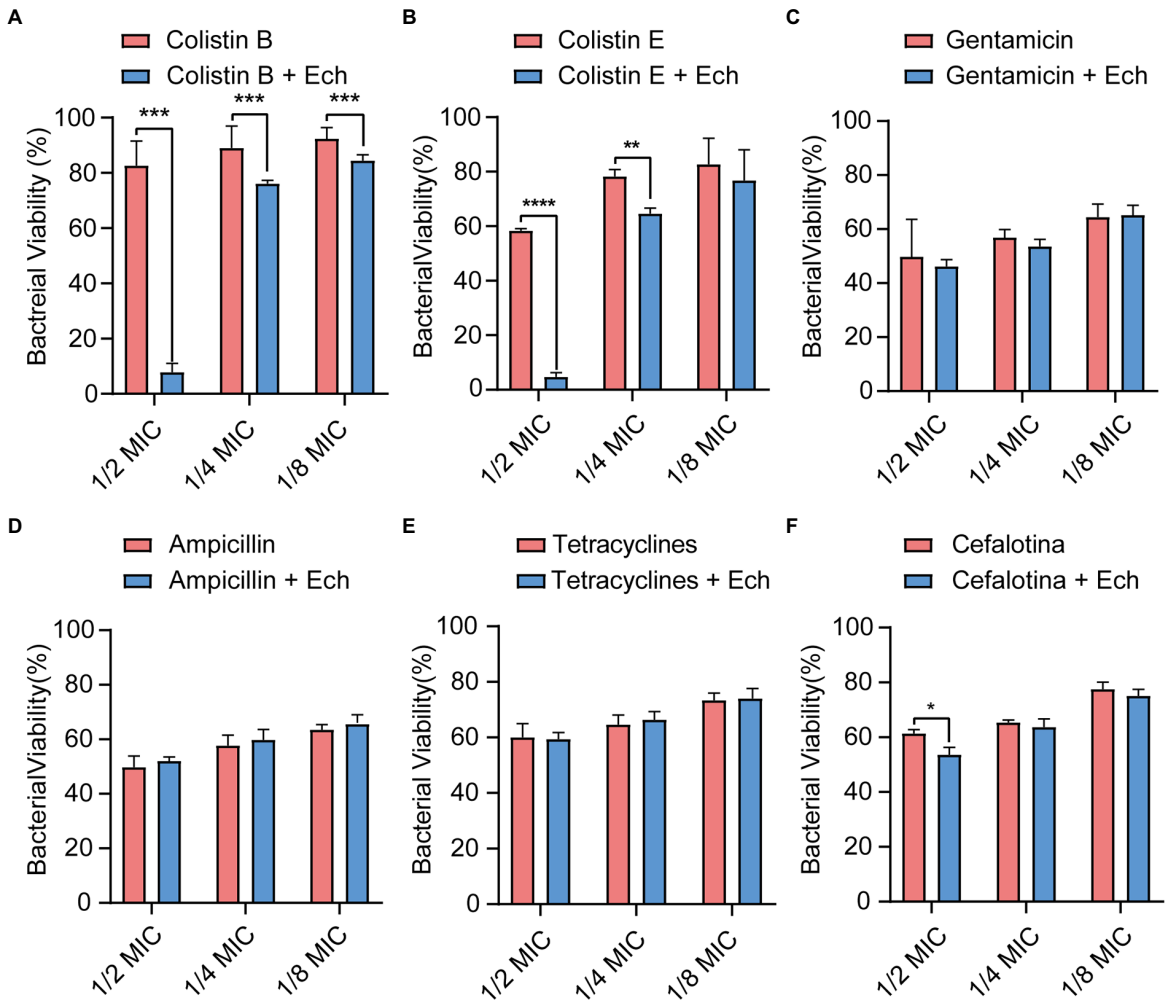
Synergistic effects of Ech (50 μM) with six different antibiotics in *E. coli* O157:H7 on bacterial viability. Data represent means ± SD of three experiments conducted in triplicate. * = *p* < 0.05; ** = *p* < 0.01; *** = *p* < 0.001;**** = *p* < 0.0001.

**Figure 9:**
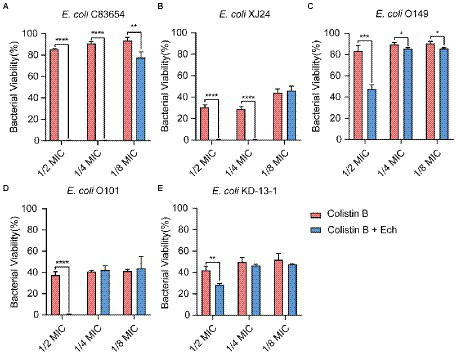
Synergistic effects of Ech (50 μM) with Colistin B in five different *E. coli* on bacterial viability. *E. coli* **(A)** C83654, **(B)** XJ24, **(C)** O149, **(D)** O101, **(E)** KD-13-1. Data represent means ± SD of three experiments conducted in triplicate. * = *p* < 0.05; ** = *p* < 0.01; *** = *p* < 0.001; **** = *p* < 0.0001.

**Figure 10:**
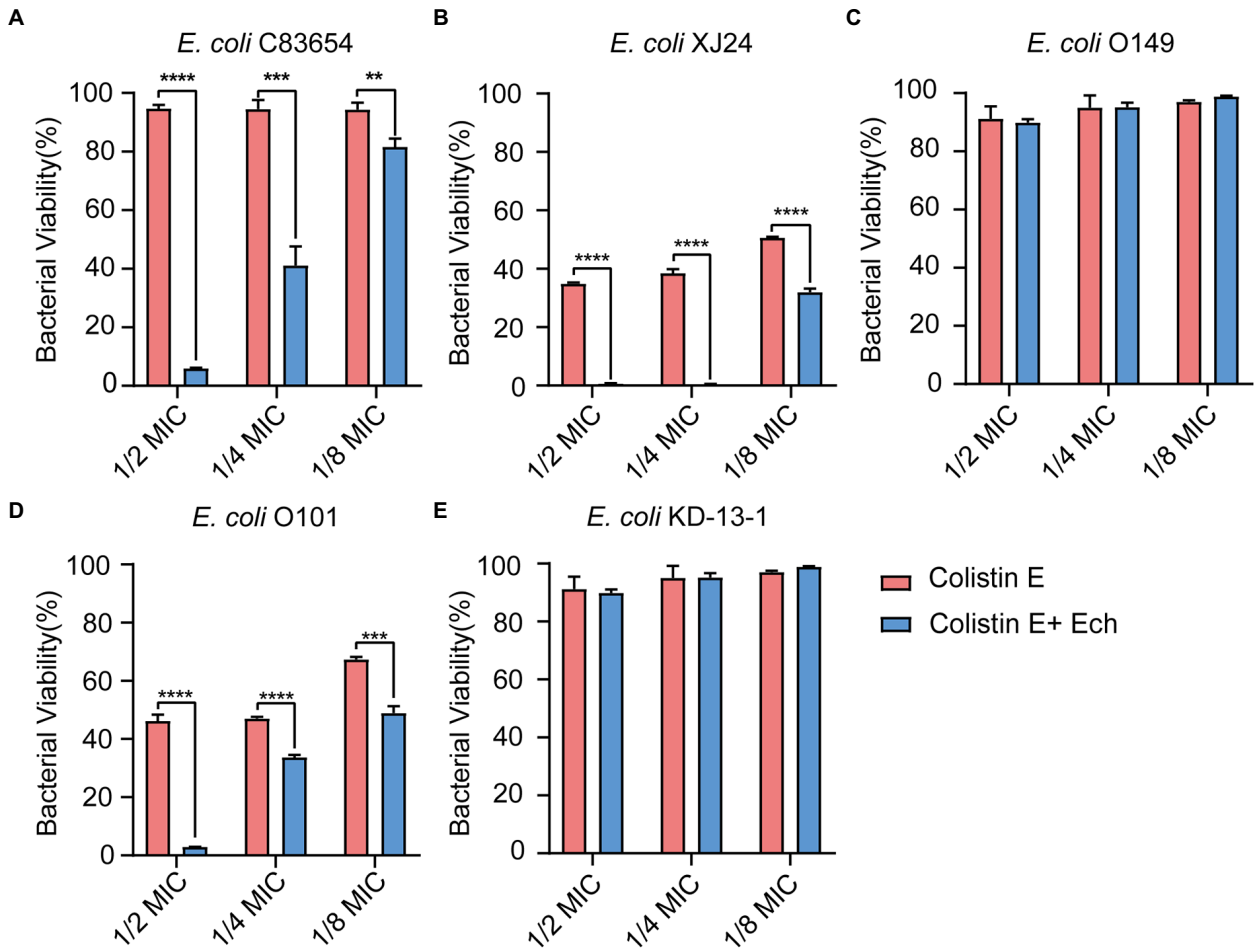
Synergistic effects of Ech (50 μM) with Colistin E in five different *E. coli* on bacterial viability. *E. coli* **(A)** C83654, **(B)** XJ24, **(C)** O149, **(D)** O101, **(E)** KD-13-1. Data represent means ± SD of three experiments conducted in triplicate. ** = *p* < 0.01; *** = *p* < 0.001; **** = *p* < 0.0001.

It was reported that antibiotic resistance was closed related to QS. So, we evaluated the synergistic antimicrobial effect of QS inhibitor Ech with conventional antibiotics against *E. coli* O157:H7 and five clinical isolates of strains (*E. coli* C83654, *E. coli* XJ24, *E. coli* O101, *E. coli* O149, *E. coli* KD-13-1). First, we evaluated the synergistic antibacterial effect of Ech and six different antibiotics against *E. coli* O157:H7. According to Figure 8, QS inhibitor Ech only has synergistic antibacterial activity with colistin antibiotics (1/2 MIC, 1/4 MIC, 1/8 MIC). In addition, the synergistic antibacterial effect of Ech with polymyxin B and polymyxin E on five clinical isolates of *E. coli* was further verified (Figures 9, 10). These results suggested that QS inhibitor Ech in conjunction with conventional antibiotics could be an efficient therapeutic strategy for inhibiting pathogens like *E. coli.*

## Discussion

Microbial infections continue to pose a serious problem because of antibiotic resistance, making alternative therapies imperative. QS not only regulates different pathogenic processes like virulence production (Defoirdt, 2018), biofilm formation (Rickard et al., 2006; Irie and Parsek, 2008), and antibiotic sensitivity (Stenvang et al., 2016; Liu et al., 2021) but also does not result in the development of resistance (Sully et al., 2014; Quave et al., 2015; Ning et al., 2021). It has emerged as the potential target for fighting antibiotic resistance (Suga and Smith, 2003; Geske et al., 2005; Rasmussen and Givskov, 2006). In the present study, we identified that Ech, a novel QS inhibitor selected from 13 different flavonoids, did not inhibit *E. coli* growth and metabolic activity but affected QS-regulated processes like biofilm formation, *EPS* production, motility, and virulence factor production. Furthermore, we demonstrated the synergistic effect of Ech combination with colistin B and colistin E against *E. coli* O157:H7 and five clinical isolates of strains. In short, we concluded that Ech presents excellent anti-QS and synergistic antibacterial effects against *E. coli*.

**Figure 11:**
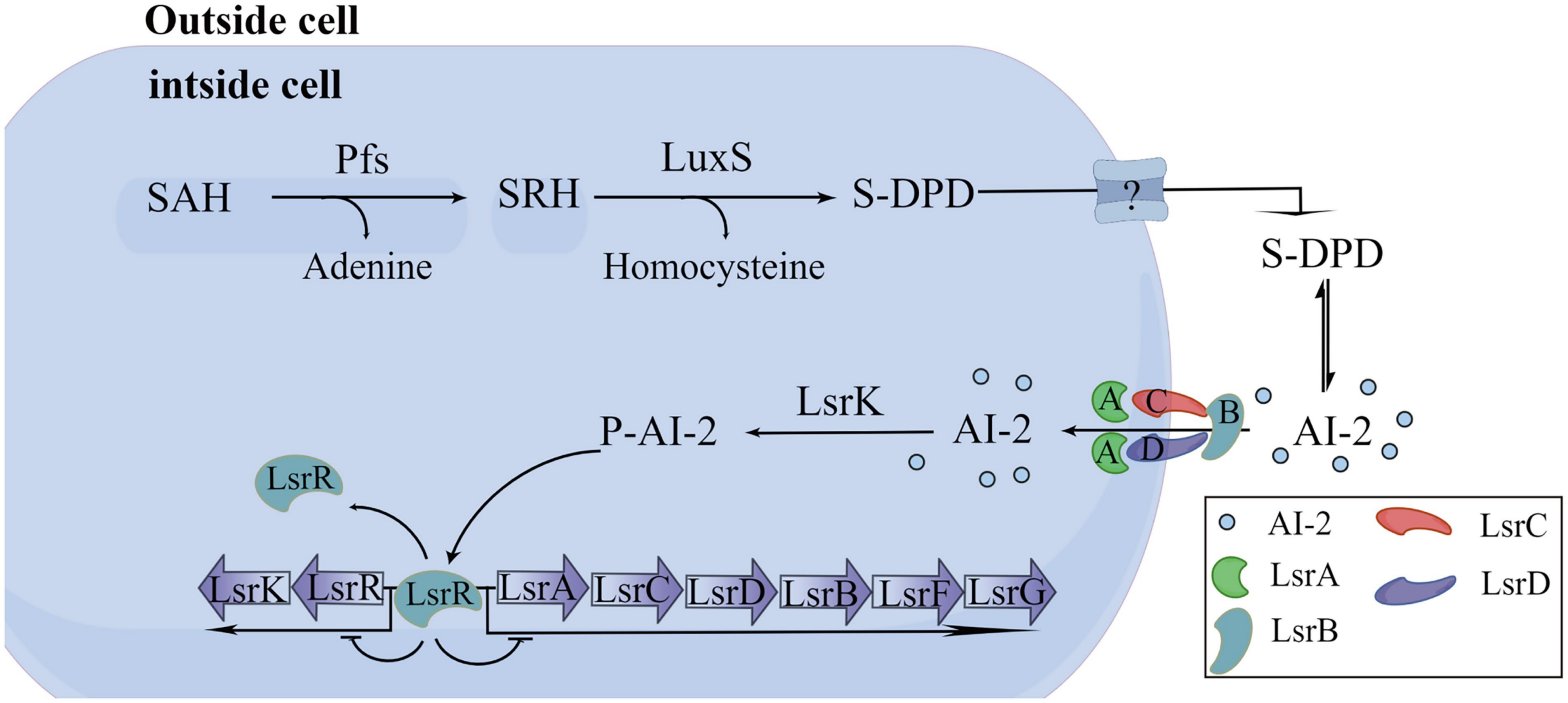
Biosynthesis, and transport of AI-2 in *E. coli*.

At present, the detection methods of signal molecule AI-2 mainly include *V. harveyi* BB170 bioluminescence assay, LuxP-based fluorescence resonance energy transfer (LuxP-FRET) reporting system assay, QS engineering protein detection assay, Gas Chromatography-Mass Spectrometer (GC–MS) assay, high-performance liquid chromatography with tandem mass spectrometric (HPLC-MS/MS) assay, and high-performance liquid chromatography with fluorescence detection (HPLC-FLD) assay (Yan et al., 2016). Among them, *V harveyi* BB170 bioluminescence assay is the most commonly used method at present. In this study, we screened Ech with high-efficiency anti-QS from 13 different flavonoids through the AI-2 bioluminescence assay. In addition, we proved that Ech had no cell toxicity in Caco-2 in the effective dose and adverse effects on bacterial growth and metabolic activity. Further, Ech also significantly decreased the expression of QS-regulated genes, including *luxS*, *pfs*, *lsrB*, *lsrK*, and *lsrR*. Several studies have shown that AI-2 regulates the formation of biofilm, the production of virulence factors, motility, and resistance of bacteria. So, pathogen virulence could be reduced by inhibiting AI-2 production (Ma et al., 2017; Sedlmayer et al., 2021; Jiang et al., 2022). *LuxS* and *pfs* are responsible for the synthesis of AI-2 with S-adenosylhomocysteine (SAH) as substrate (Figure 11; Zhao et al., 2018; Wu et al., 2019). Similarly, it has been reported that *luxS* and *pfs* deletion mutants of *E. coli* inhibit AI-2 production, biofilm formation, and motility (Yang et al., 2014; Wang et al., 2016; Zuberi et al., 2017). Therefore, the *luxS* and *pfs* have been considered important therapeutic drug targets, and many inhibitors against *luxS* and *pfs* have been investigated (Shivaprasad et al., 2021; Sharifi and Nayeri Fasaei, 2022). The *lsr* system is liable for AI-2 detection, uptake, and signal transduction in *E. coli* (Pereira et al., 2013). *LsrR* is an inhibitor of the *lsr* system. There is very little extracellular AI-2 during the early stages of bacterial growth. *LsrR* is active and inhibits the transcription of the *lsr* system. *LsrK* phosphorylates uptake of AI-2 as it accumulates in the extracellular space. The binding of phospho-AI-2 to *lsrR* results in the initiation of transport by the *lsr* system. *LsrB* is an AI-2 receptor responsible for the internalization of AI-2 in *E. coli* (Figure 11). Similarly, *lsrR*, *lsrK*, and *lsrB* deletion mutant has been reported to interfere with signal transduction and inhibit biofilm, motility, and pathogenicity (Li et al., 2007; Jani et al., 2017; Laganenka and Sourjik, 2018; Zuo et al., 2019). So, the *lsrR*, *lsrK*, and *lsrB* have been considered important therapeutic drug targets. Many inhibitors against *lsrR, lsrK, and lsrB* have been investigated (Peng et al., 2018; Gatta et al., 2019; Stotani et al., 2019; Gatta et al., 2020). Our results suggested that Ech could interfere with the AI-2 synthesis, secretion, or transport through their effects on *luxS*, *pfs*, *lsrK*, or *lsrB*.

The biofilm is an aggregate of microorganisms where microorganisms are adhered to the substrate (e.g., stainless, glass, meats, and vegetables) and encapsulated within a self-produced matrix of *EPS*, proteins, and extracellular DNA (eDNA) (Hobley et al., 2015; Flemming et al., 2016; Karygianni et al., 2020). *EPS* can separate colonies and form a pathway for transporting metabolites and nutrients. In addition, *EPS* can maintain the biofilm’s structure and contribute to bacteria colonization on the non-biological surface (Fan et al., 2022). Curli fimbriae and cellulose, whose products are regulated by *csgD*, are common *EPS* components found in bacterial biofilms (Dieltjens et al., 2020). *CsgD* stimulates the formation of *E. coli* biofilms by simultaneously activating the expression of the curli- encoding genes *csg* operon and inhibiting negative affect factors such as *yagS* and *pepD* (Brombacher et al., 2003). Otherwise, *csgD* directly inhibits flagellum formation of *fliE* and *fliEFGH* operon, thereby regulating biofilm formation and cell motility by inhibiting flagellum formation and rotation. This study showed that Ech inhibited *EPS* production in a dose-dependent manner by Ruthenium Red stain. Further, Ech also significantly decreased *csgD* expression (Figure 7). The results demonstrated that Ech reduced *EPS* production by inhibiting QS.

We performed motility experiments in a semi-solid agar medium to determine whether Ech affects bacterial motility. The results showed that Ech inhibited bacterial motility in a dose-dependent manner. Additionally, flagellum-regulated genes such as *flhC*, *flhD*, and *fliC* were also significantly down-regulated by Ech. *FlhDC* is an activator of the flagellum regulatory cascade, which regulates flagellum synthesis and motility (Guttenplan and Kearns, 2013). *FliC*, as a flagellum filament structural protein, is involved in the pathogenesis of infection, the production of biofilm, and motility (Chaban et al., 2015). The results demonstrated that Ech reduced flagellum motility by inhibiting QS.

Substances that constitute bacterial virulence are called virulence factors including invasiveness and virulence. Among the six major pathotypes of *E. coli*, Shiga toxin-producing *E. coli* is the most prevalent (Moreau et al., 2021). Shiga toxin is one of the major virulence factors of Shiga toxin-producing *E. coli* O157: H7. It can induce the formation of A/E lesions, and cause HUS if it enters the circulatory system (Kordbacheh et al., 2019). In humans, the stx2 gene codes for the Stx2 which is associated with more severe diseases caused by Shiga toxins, which play an essential role in the pathogenesis of *E. coli* O157:H7 (Sheng et al., 2016). In the present student, we found that the virulence factor regulatory gene stx2 was downregulated by 69%. The results demonstrated that Ech reduced virulence factor production by inhibiting QS.

Multiple antibiotic resistance mechanisms have evolved due to the extensive use of antibiotics during clinical treatment. The primary resistance mechanisms are to passivate antibiotics by eliminating antibiotics by efflux pump system, chemical modifications, and target gene modification (Zhao et al., 2020). Meanwhile, the biofilms formed by many pathogens result in strong resistance (Rajput et al., 2018). QS system can reduce antibiotic resistance by regulating biofilm formation (Zhao et al., 2020). In this study, we found that combined QS inhibitor Ech and colistin antibiotics could play a synergistic antibacterial effect. The results suggested that combining antibiotics with anti-QS compounds appears to be an effective therapeutic strategy for treating pathogen infections.

## Data availability statement

The original contributions presented in the study are included in the article/Supplementary material, further inquiries can be directed to the corresponding author.

## Author contributions

Y-BB wrote the manuscript and participated in some experiments. M-YS, L-YW, and Y-TB performed part of the experiments. W-WW, X-ZZ, and BL revised the manuscript. J-YZ and W-WW directed the project and reviewed the manuscript. All authors contributed to the article and approved the submitted version.

## Funding

This research was funded by the research on the National Natural Science Foundation of China, grant number 32102727–the earmarked fund for CARS, grant number CARS-37–Innovation Project of Chinese Academy of Agricultural Sciences, grant number 25-LZIHPS-05.

## Conflict of interest

The authors declare that the research was conducted in the absence of any commercial or financial relationships that could be construed as a potential conflict of interest.

## Publisher’s note

All claims expressed in this article are solely those of the authors and do not necessarily represent those of their affiliated organizations, or those of the publisher, the editors and the reviewers. Any product that may be evaluated in this article, or claim that may be made by its manufacturer, is not guaranteed or endorsed by the publisher.
