# Supplementary material for: Novel quorum sensing inhibitor Echinatin as an antibacterial synergist against Escherichia coli
Source: Front Microbiol. 2022 Nov 1;13:1003692. doi: 10.3389/fmicb.2022.1003692 (PMC9663819; doi:10.3389/fmicb.2022.1003692)
Supplement: Supplementary file 1 [file Data_Sheet_1.PDF]

Supplementary Table 1. Primer sequences used for qRT-PCR amplification

Genes		sequence (5' to 3')	Product size (bp)
gapA	F	CCAGGACATCGTTTCCAAC	103
gapA	R	GGTGGTCATCAGACCTTCG	103
csgD	F	TGATGAACAACGAACGAGCGATCTC	146
csgD	R	GCTTGCCAGTTACCTGATTACACATTC	146
flhC	F	ATGCTGCCATTCTCAACCGACTG	117
flhC	R	CGCATCGACGCCATTACACAAAC	117
flhD	F	CGTTAGCGGCACTGACTCTTCC	107
flhD	R	TTGCGTCAACTGAGTAATCGTCTGG	107
fliC	F	TTACCAACCTGAACAACACCACTACC	90
fliC	R	ACATATTGGACACTTCGGTCGCATAG	90
luxS	F	GAAAACAATGAACACCCCGCATGG	92
luxS	R	TCCCTCTTTCTGGCATCACTTCTTTG	92
pfs	F	GTAGAGATGGAAGCGACGGCAATC	129
pfs	R	AACAGCCAGGAACTCATCGAAGC	129
lsrB	F	AGTGCTGACCTGGGACTCTGATAC	99
lsrB	R	GCCATATCCACCAACATACCTCCTAAC	99
lsrK	F	GATGAACCTACCGCCTCGCTTAC	90
lsrK	R	AACAATACCCACGCCAGTAGCAAG	90
lsrR	F	ACCACAACAGATGCTGGCGATTG	143
lsrR	R	GCTGCCCGATTCCCGTCATATAAG	143
stx2	F	CTTACGCTTCAGGCAGATACAGAGAG	261
stx2	R	TCTTCATTCACGGCGCGAACAG	261
