# Supplementary material for: Novel quorum sensing inhibitor Echinatin as an antibacterial synergist against Escherichia coli
Source: Front Microbiol. 2022 Nov 1;13:1003692. doi: 10.3389/fmicb.2022.1003692 (PMC9663819; doi:10.3389/fmicb.2022.1003692)
Supplement: Supplementary file 2 [file Data_Sheet_2.PDF]

Supplementary Table 2. Screening results of QS inhibitors

Compound	Inhibition（%）	IC50(μM)
Echinatin	77.08	21.76
Aloeemodin	74.90	40.07
Loureirin B	64.82	43.95
Phloretin	62.52	45.24
Cardamonin	62.00	46.16
Neosperidin dihydrochalcone	61.34	46.74
Cynaroside	60.77	46.32
Acacetin	60.01	47.63
Scutellarin	45.44	-
Artemetin	40.78	-
Hesperetin	38.75	-
Vitexin	38.39	-
Gentiin	21.26	-

Note: “-” represent not assayed.
